# Efficacy of antibiotic therapy for peritoneal dialysis-associated peritonitis: a proportional meta-analysis

**DOI:** 10.1186/1471-2334-14-445

**Published:** 2014-08-18

**Authors:** Pasqual Barretti, João Vitor Pereira Doles, Douglas Gonçalves Pinotti, Regina El Dib

**Affiliations:** Botucatu Medical School, UNESP - Universidade Estadual Paulista, São Paulo, Brazil

**Keywords:** Peritonitis, Peritoneal dialysis, Treatment, Meta-analysis

## Abstract

**Background:**

The choice of antimicrobials for initial treatment of peritoneal dialysis (PD)-related peritonitis is crucial for a favorable outcome. There is no consensus about the best therapy; few prospective controlled studies have been published, and the only published systematic reviews did not report superiority of any class of antimicrobials. The objective of this review was to analyze the results of PD peritonitis treatment in adult patients by employing a new methodology, the proportional meta-analysis.

**Methods:**

A review of the literature was conducted. There was no language restriction. Studies were obtained from MEDLINE, EMBASE, and LILACS. The inclusion criteria were: (a) case series and RCTs with the number of reported patients in each study greater than five, (b) use of any antibiotic therapy for initial treatment (e.g., cefazolin plus gentamicin or vancomycin plus gentamicin), for Gram-positive (e.g., vancomycin or a first generation cephalosporin), or for Gram-negative rods (e.g., gentamicin, ceftazidime, and fluoroquinolone), (c) patients with PD-related peritonitis, and (d) studies specifying the rates of resolution. A proportional meta-analysis was performed on outcomes using a random-effects model, and the pooled resolution rates were calculated.

**Results:**

A total of 64 studies (32 for initial treatment and negative culture, 28 reporting treatment for Gram-positive rods and 24 reporting treatment for Gram-negative rods) and 21 RCTs met all inclusion criteria (14 for initial treatment and negative culture, 8 reporting treatment for Gram-positive rods and 8 reporting treatment for Gram-negative rods). The pooled resolution rate of ceftazidime plus glycopeptide as initial treatment (pooled proportion = 86% [95% CI 0.82–0.89]) was significantly higher than first generation cephalosporin plus aminoglycosides (pooled proportion = 66% [95% CI 0.57–0.75]) and significantly higher than glycopeptides plus aminoglycosides (pooled proportion = 75% [95% CI 0.69–0.80]. Other comparisons of regimens used for either initial treatment, treatment for Gram-positive rods or Gram-negative rods did not show statistically significant differences.

**Conclusion:**

We showed that the association of a glycopeptide plus ceftazidime is superior to other regimens for initial treatment of PD peritonitis. This result should be carefully analyzed and does not exclude the necessity of monitoring the local microbiologic profile in each dialysis center to choice the initial therapeutic protocol.

**Electronic supplementary material:**

The online version of this article (doi:10.1186/1471-2334-14-445) contains supplementary material, which is available to authorized users.

## Background

Although continuous peritoneal dialysis (PD) was introduced almost four decades ago, its application continues to be hindered by peritonitis, despite the large reduction of peritonitis incidence due to advances in connectology and widespread use of antibiotic prophylaxis. Peritonitis remains as a serious complication influencing patients’ mortality, and is the most frequent cause of PD failure [1].

The choice of antimicrobial therapy for initial treatment is a crucial determinant for a favorable clinical course and outcome. Historically, this choice has been based on the recommendations of the International Society for Peritoneal Dialysis (ISPD), which has published six documents between 1989 and 2010 [2–7]. According to these guidelines, the initial treatment of peritonitis (prior to the results of microbiological tests) should be based on associations of drugs for coverage of Gram-positive cocci and Gram-negative bacilli. The recommendations about the class of antimicrobials have varied over time. In general, for coverage of Gram-positive cocci the use of a first generation cephalosporin or vancomycin has been proposed, while for Gram-negative bacilli an aminoglycoside or ceftazidime have been recommended. However, based on the available literature there is no consensus about the best antimicrobial therapy for the initial treatment of these infections, and few prospective and controlled studies have been published.

A systematic review with a meta-analysis of randomized controlled trials, published by Wiggins et al. [8], included 36 studies published between 1985 and 2006, and did not report superiority of any class of antimicrobials. One limitation of the study was the exclusion of a large number of publications with a high number of patients and episodes of peritonitis. Most of these excluded studies were case series. Thus, the present study aimed to analyze the clinical results of PD related peritonitis treatment reported in both, randomized controlled trials (RCTs) and case series studies employing an alternative methodology, the proportional meta-analysis, and to examine possible differences among therapeutic protocols.

## Methods

### Literature search and studies selection

A review of case series and RCTs containing the treatment of PD-related peritonitis was performed. There was no language restriction. Studies were obtained from the following sources: US National Library of Medicine (PUBMED; 1966–2013), Excerpta Medica database (EMBASE; 1980–2013) and Literatura Latino-Americana and Caribe em Ciências da Saúde (LILACS; 1982–2013). The last search date was 11^th^ January, 2013.

The databases were examined using a comprehensive search strategy for PD-related peritonitis and antibiotic therapy, along with MeSH and text words, including a list of synonyms (Appendix). The search strategy was adapted for each database in order to maximize the ability to identify eligible studies. The bibliographic references in relevant articles were also examined for eligible studies.

The following inclusion criteria were used: (a) RCTs and case series studies with a number of reported patients greater than five, (b) use of any antibiotic therapy, regardless of whether it was used for initial treatment (e.g., cefazolin plus gentamicin or vancomycin plus gentamicin), for Gram-positive rods (e.g., vancomycin or a first generation cephalosporin), or for Gram-negative rods (e.g., gentamicin, ceftazidime, and fluoroquinolone), (c) patients with PD-related peritonitis, and (d) studies specifying the rates of peritonitis resolution. The data from RCTs were incorporated in the analysis as discrete data sets. Studies in pediatric patients and those with incomplete data were excluded from the review.

Peritonitis diagnosis was based on at least two of the following: abdominal pain or cloudy dialysate, dialysate white cell count >100/µL with at least 50% neutrophilic cells, and positive culture of dialysate [6, 7]. We defined peritonitis resolution based on the following definitions used by authors of the included studies: disappearance of signs and symptoms within 96 h after the beginning of antibiotic therapy and a negative peritoneal fluid culture at least 28 days after treatment completion; an episode of peritonitis where the catheter remained *in situ* and symptoms and signs resolved; initial response to antibiotic therapy combined with no need to remove the PD catheter; complete resolution of peritonitis without relapse for 30 days following initial therapy completion; absence of symptoms of peritonitis and clear dialysate effluent 5 days after start of antibiotic therapy; sterilization of the dialysate with no relapse within 4 weeks after treatment; no relapse within 2 weeks after ceasing treatment; cure without altering either of the empirical antibiotics to second-line antibiotics; resolution of abdominal pain, clearing of dialysate, and dialysate neutrophil count less than 100/µL on day 10; complete resolution of peritonitis by antibiotics alone without relapse or recurrence within 4 weeks of completion of therapy; PD fluid became clear, patient survived the period of the treatment of peritonitis and 4 weeks after treatment ceased; PD catheter did not require removal to clear the infection, and no relapse of peritonitis caused by the same organism or with negative culture results within 4 weeks post treatment of the initial episode [6, 7].

### Data collection

Two reviewers independently screened the titles identified by the literature search, extracted the data from the studies, and analyzed the results. Discrepancies in the results were resolved by discussion by the reviewers. A standard form was used to extract the following information: authors and year of publication, country, number of participants and peritonitis episodes, patients’ mean age, basal renal disease, comorbidities, PD modality (continuous ambulatory peritoneal dialysis [CAPD] or automated peritoneal dialysis [APD]), initial peritonitis treatment protocol and its adjustments, and outcomes.

We used the risk of bias approach for Cochrane Reviews to assess the RCT quality [9] as we are used to critical appraise RCT with this tool. Please, find below the reference. We have included one figure entitled Risk of bias summary: review authors’ judgments about each risk of bias item for each RCT included.

### Statistical analysis

The outcomes were treated as a dichotomous variable (peritonitis resolution versus no resolution) with respective 95% confidence intervals (CI). Statistical heterogeneity was assessed with the I^2^ statistic, and significance was assumed when the I^2^ was greater than 50%. The I^2^ statistic illustrates the percentage of the variability in effect estimates resulting from heterogeneity rather than sampling error [10, 11]. Because of the clear differences among the included studies and several uncontrolled variables, we used a random-effect model [12] to perform a proportional meta-analysis of case series studies [13, 14]. The software used to plot the studies in the meta-analysis was StatsDirect.

For first generation cephalosporins, we included: cefazolin, cephalotin, cefamezin and cephaloridine. The only third generation cephalosporin we analyzed was ceftazidime. For aminoglycosides we included gentamicin, amikacin, netilmicin and tobramycin. Vancomycin and teicoplanin were considered in the analysis as glycopeptides. Finally, ciprofloxacin, levofloxacin and ofloxacin were the fluoroquinolones included.

A statistically significant difference between interventions was defined when their combined 95% CIs did not overlap [13, 14]. We considered p < 0.05 as statistically significant.

## Results

The literature search was conducted through January 2013, and 6,743 titles had been identified. After the screening by title and abstract, we obtained full paper copies of 140 studies reporting antibiotic therapy for PD-related peritonitis that were eligible for inclusion. However, 56 of these studies were either cohort or off-topic. Hence, only a total of 64 case series studies (32 reporting initial treatment and negative culture, 28 reporting treatment for Gram-positive rods and 24 reporting treatment for Gram-negative rods) and 20 RCTs met all inclusion criteria (14 for initial treatment and negative culture, eight reporting treatment for Gram-positive rods and eight reporting treatment for Gram-negative rods). These studies included 9.268 patients with 16.109 episodes of peritonitis. A total of 4.411 patients (7.315 episodes) were reported for the initial treatment and negative culture, 3.526 patients (6.259) were reported for the Gram-positive group, and 2.549 (4.925) were reported for the Gram-negative group (Table 1).

**Table 1:** **Characteristics of case series and RCT studies including in the qualitative analysis, according to treatment target (initial, gram-positive and gram-negative rods) and the patient’s renal basal disease**

Study	All studies	Initial treatment/Negative culture	Gram +	Gram -
**Total of studies (case series and RCTs)**	84 [15-98]	44 [15-24,26,28,34,40,55,57,60-87]	36 [15,18,20-25,29,30,33-37,42,44,48,50,54,55,57,63,75,77,85,86,88-95]	32 [15,18,20,22,23,25,,31,32,35-37,39,41,45-47,49,50,52,55,57,63,69,77,78,86,91,93,96-98]
**No. of patients/No. of episodes**	9.268/16.109	4.411/7.315	3,526/6,259	2,549/4,925
**Basal renal disease**				
Branchio-oto-renal syndrome	1	0	0	0
Chronic tubulointerstitial disease	2	1	1	0
Diabetes	51	16	14	10
Glomerulonephritis	33	12	10	6
Gouty	1	1	0	0
Hemolytic-uremic syndrome	1	0	0	0
Hypertension	21	13	8	5
IgA nephropathy	1	1	0	0
Interstitial nephritis	3	1	0	0
Systemic lupus	5	2	1	1
Malignancy	2	0	1	0
Multiple myeloma	1	0	0	0
Nephrosclerosis	2	2	2	0
Obstruction/Reflux	15	5	4	4
Others/unknown	25	11	8	3
Pyelonephritis	4	3	1	1
Policystic kidney disease	24	8	8	5
Renal artery stenosis	1	1	0	0
Renovascular	12	3	1	1
Systemic autoimmune disease	2	2	1	0
**Comorbidities**				
AIDS	1	1	0	0
Cerebrovascular disease	7	1	2	0
Chronic lung disease	7	1	3	1
Connective tissue disorder	1	1	1	0
Congestive heart failure	2	1	1	0
Coronary heart disease	9	1	4	1
Current smoker	4	0	1	0
Dementia	2	1	1	0
Diabetes	16	6	8	5
Hemiplegia	2	1	1	0
Mild liver disease	2	1	1	0
Moderate or severe liver disease	3	2	1	0
Peptic ulcer disease	2	1	1	0
Peripheral vascular disease	9	1	2	0
Secondary hyperparathyroidism	1	0	1	1
Any Tumor, Leukemia, Lymphoma	4	2	3	1
**Type of dialysis**				
**CAPD**	50	32	24	23
**APD**	11	7	7	7
**Not reported**	37	9	11	9
**Any change from APD to CAPD**	NR	NR	NR	NR
**Mean age (years)**	57,36	55,65	58,44	56,53

NR = not reported; CAPD = continuous ambulatory peritoneal dialysis; APD = automated peritoneal dialysis; RCT = randomized clinical trial.

**Figure 1:**
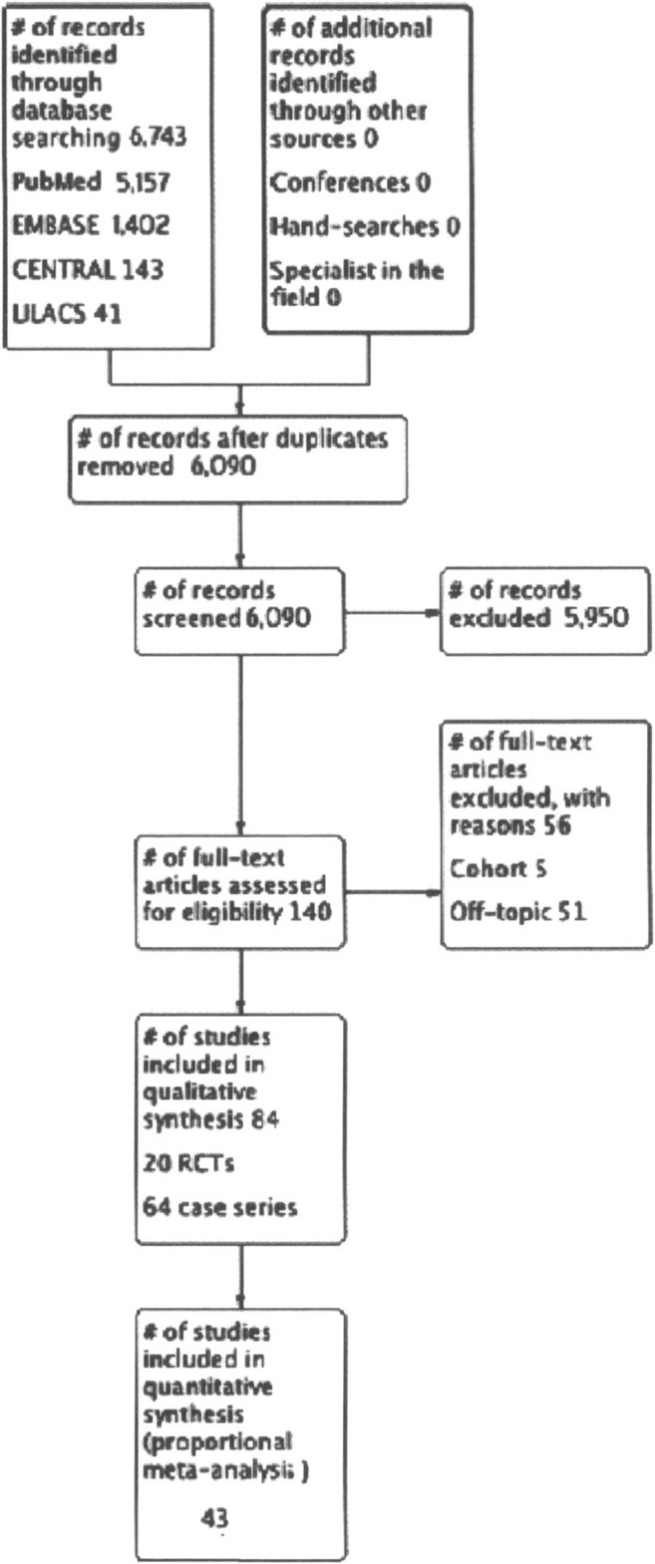
**Study flow diagram.**

**Figure 2:**
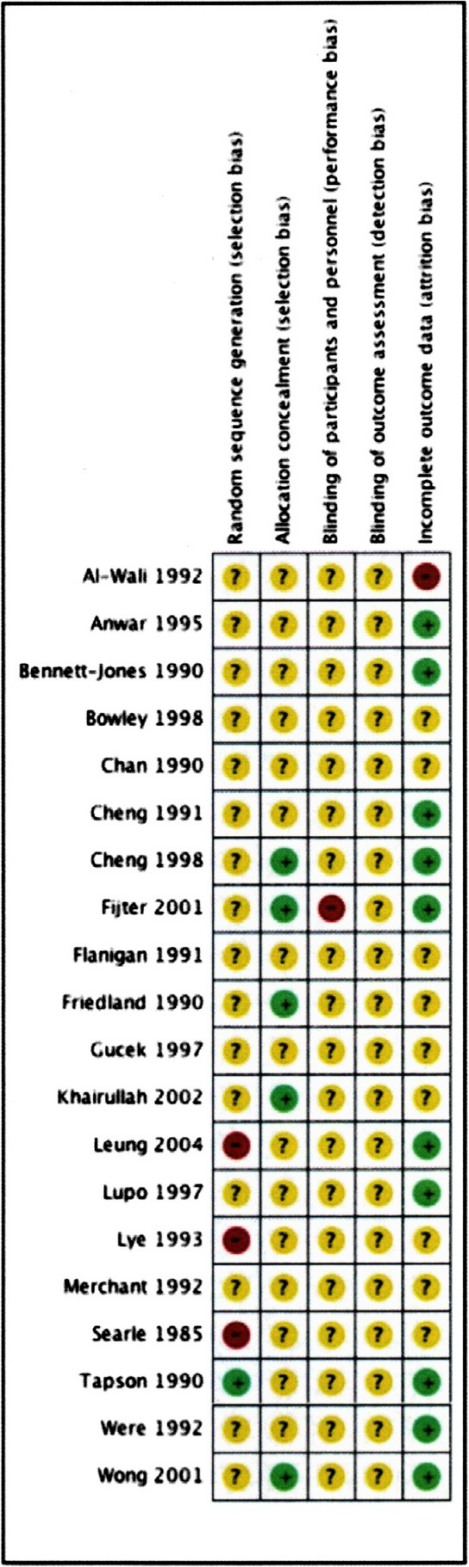
**Risk of bias summary of randomized control trials: review authors' judgments about each risk of bias item for each included study.**

However, from these total, 38 case series [15–52] were not included in the meta-analysis due to the lack of data. Methodological aspects of five RCT studies [53–57] had a risk of introducing bias, with inadequate blinding of participants, random sequence generation and incomplete outcome, and three RCTs was excluded from the quantitative analysis due to lack of data [53, 56, 58]. In this way, proportional meta-analysis was performed from 43 studies (Figure 1). We have summarized the risk of bias of RCT included studies in Figure 2.

### Comparisons for initial treatment or culture negative episodes

**Figure 3:**
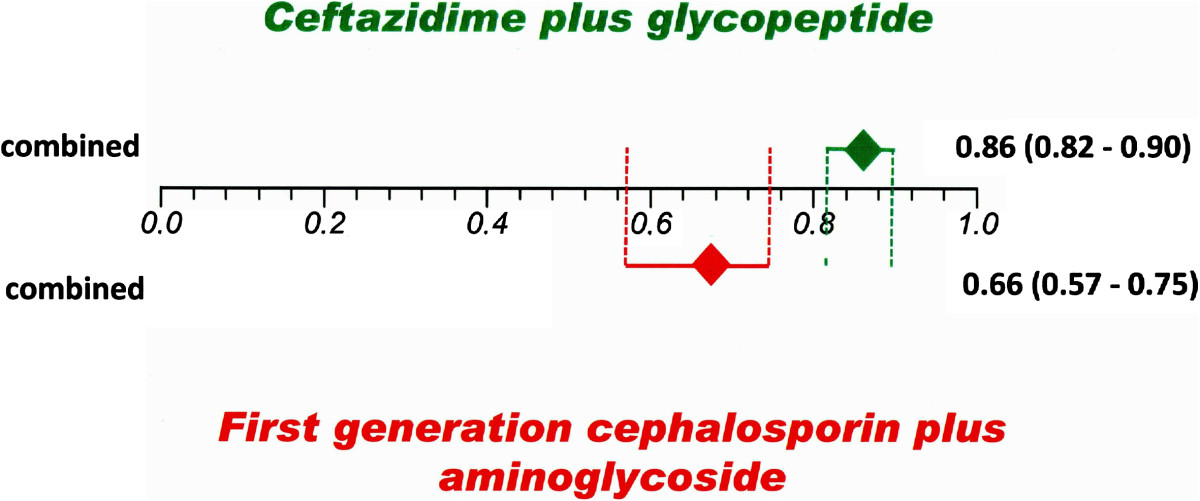
**Combined resolution rate with 95% CIs of studies of initial treatment with ceftazidime plus a glycopeptide versus a first generation cephalosporin plus an aminoglycoside.**

**Figure 4:**
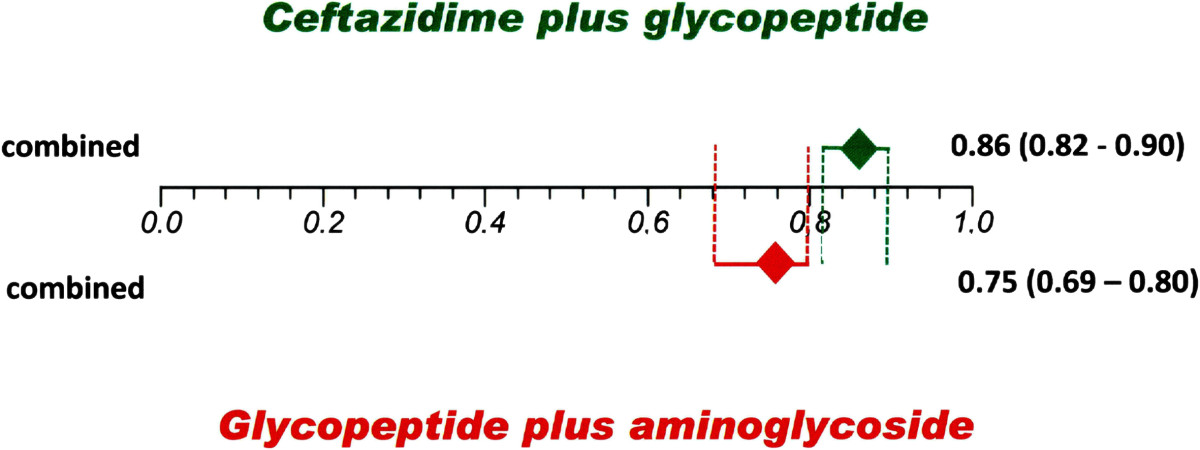
**Combined resolution rate with 95% CIs of studies of initial treatment with ceftazidime plus a glycopeptide compared to a glycopeptide plus an aminoglycoside.**

Ceftazidme plus a glycopeptide as initial treatment was used in five studies [59–63] with 443 episodes; the pooled resolution rate was 86% (95% CI 0.82–0.89). This resolution rate was significant higher than initial treatment with a first generation cephalosporin plus aminoglycosides (pooled proportion of 66%, 95% CI 0.57–0.75) from 14 included studies [57, 61, 64–75] with 1,438 total episodes (Figure 3). Initial treatment with ceftazidime plus a glycopeptide also showed a higher resolution rate than a glycopeptide plus aminoglycosides (pooled proportion of 75%, 95% CI 0.69–0.80) that were used in 16 included studies [55, 66–68, 75–86] with 574 episodes (Figure 4).

The following comparisons did not show statistically significant differences because their CIs overlapped: a first generation cephalosporin plus aminoglycosides (resolution rate = 66%, 95% CI 0.57–0.75) versus glycopeptides plus aminoglycosides (resolution rate = 75%, 95% CI 0.69–0.80); a first generation cephalosporin plus aminoglycosides (resolution rate = 66%, 95% CI 0.57–0.75) versus a first generation cephalosporin plus ceftazidime (resolution rate = 59%, 95% CI 0.32–0.83); glycopeptides plus aminoglycosides (resolution rate = 75%, 95% CI 0.69–0.80) versus first generation cephalosporin plus ceftazidime (resolution rate = 59%, 95% CI 0.32–0.83), and a first generation cephalosporin plus ceftazidime (resolution rate = 59%, 95% CI 0.32–0.83) versus ceftazidime plus a glycopeptide (resolution rate = 86%, 95% CI 0.82–0.89).

There was significant heterogeneity among studies for three of the initial treatment used (ceftazidme plus glycopeptide I^2^ = 91.5%; first generation cephalosporin plus third generation cephalosporin, I^2^ = 94.8%; third generation cephalosporin plus glycopeptide, I^2^ = 8,02E-02% .

### Comparisons for episodes due to gram-positive rods

For treatment of episodes due to Gram-positive rods, the pooled resolution rate from 13 studies [54, 55, 62, 76, 84, 85, 87–93] with 917 episodes was 78% (95% CI 0.66–0.88) for a glycopeptide, while from five studies [57, 74, 88, 93, 94] with 532 episodes for a first generation cephalosporin it was 73% (95% CI 0.55–0.88). There was no significant difference between the schemes.

There was significant heterogeneity among studies for both first generation cephalosporin and glycopeptide: I^2^ = 94.6% and 94%, respectively.

### Comparisons for episodes due to gram-negative rods

**Figure 5:**
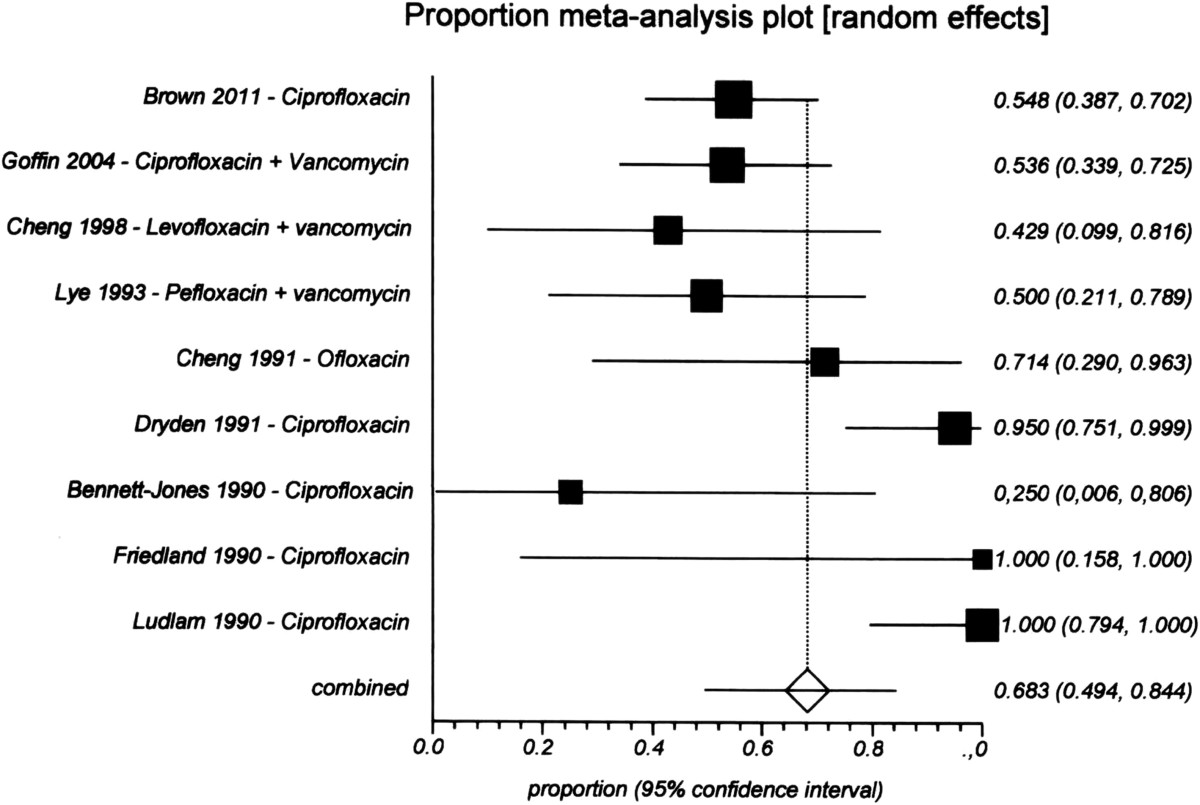
**Proportional meta-analysis of studies of the resolution rate of quinolone treatment for gram-negative peritonitis.**

**Figure 6:**
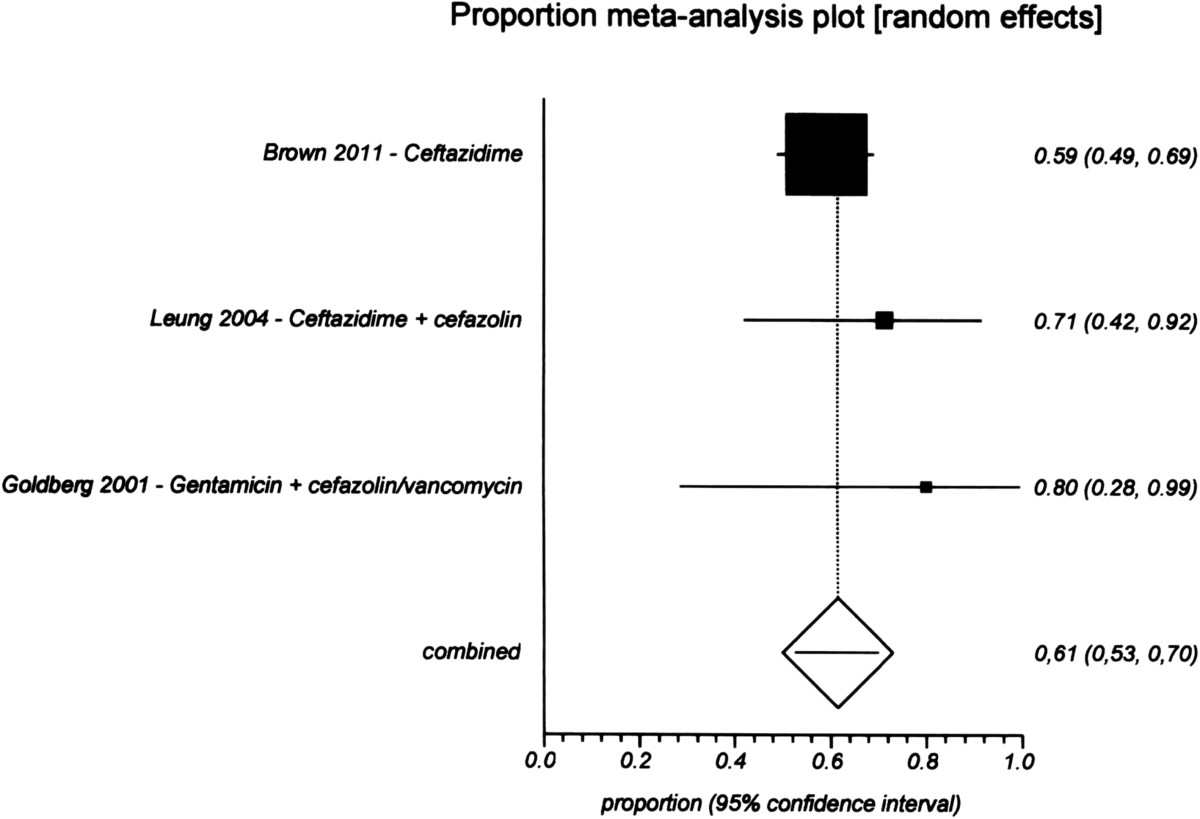
**Proportional meta-analysis of studies of the resolution rate of ceftazidime treatment for gram-negative peritonitis.**

**Figure 7:**
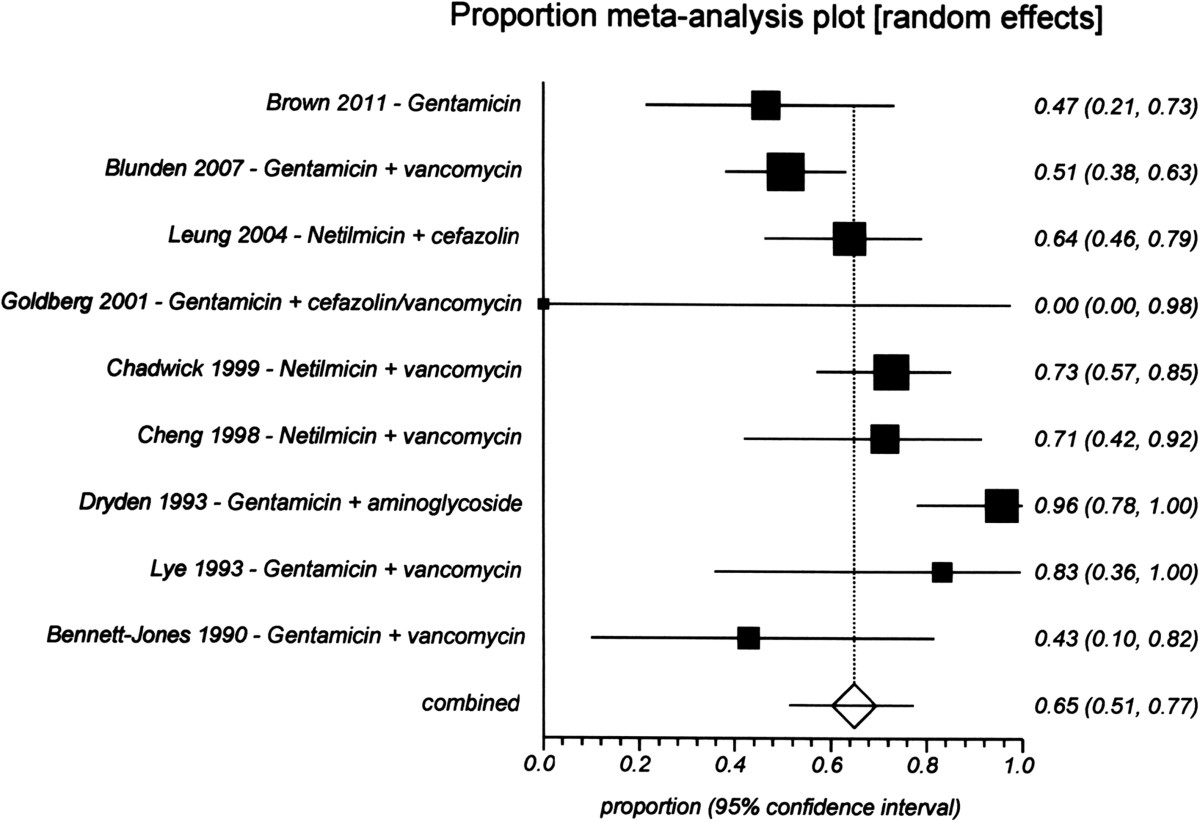
**Proportional meta-analysis of studies of the resolution rate of aminoglycosides treatment for gram-negative peritonitis.**

The pooled proportion resolution rate from nine studies [55, 76, 85, 92, 95–98] with 138 episodes was 68% (95% CI 0.50–0.85) for a quinolone (Figure 5). For ceftazidime, the resolution rate was 61% (95% CI 0.53–0.70) from three studies [68, 56, 98] with 117 episodes (Figure 6), and for aminoglycosides it was 65% (95% CI 0.51–0.77) from nine studies [55, 57, 62, 68, 76, 85, 90, 97, 98] with 211 episodes (Figure 7). There were no significant differences among the three drugs because their CIs overlapped.

There was significant heterogeneity among studies for both of the two drugs: I^2^ value was 79.3% for quinolone, and 71.1% for aminoglycosides.

## Discussion

The choice of initial treatment of PD-related peritonitis remains a challenge to nephrologists who perform PD, particularly because of the absence of evidence to indicate superiority of particular recommended therapeutic protocols. Although the only available systematic review with meta-analysis of randomized clinical trials [8], and its recent update [99] did not show superiority of a specific class of antimicrobials a review of therapeutic protocols proposed by ISPD guidelines used in case series studies (which are typically excluded from meta-analyses) could potentially show differences in outcomes among antimicrobial regimens. In addition, the possibility of performing randomized clinical trials with a sufficient number of patients has become more remote because of the current low incidence of PD-related peritonitis.

A narrative review of antimicrobial treatment of patients with PD-related peritonitis published in 1991 [100] concluded that the optimal empirical treatment was weekly vancomycin plus ceftazidime. Interestingly, the present study using proportional meta-analysis of case series was able to identify the superiority of the combination of glycopeptides plus ceftazidime in the initial treatment of PD-related peritonitis, when compared with a glycopeptide plus an aminoglycoside and when compared with a first generation cephalosporin plus aminoglycosides. This result strongly suggests that the differences found may be related to a better coverage of Gram-negative bacilli of third generation cephalosporin compared with aminoglycosides. Bacterial resistance of Gram-negative bacilli, particularly *Pseudomonas* species, to commonly prescribed antimicrobials has been reported in recent years [101]; this may explain the superiority of the protocols employing ceftazidime. We found a low-resolution rate associated with regimens based on aminoglycosides for treatment of episodes caused by Gram-negatives. It was noticeable that papers of the decade 90 presenter higher resolution rate than those published after 2000, which could result of a temporal increase of bacterial resistance to these antibiotics. In agreement, low and decreasing susceptibility rate of *Pseudomonas spp* to gentamycin was reported in our center where only 40% of strains were susceptible in the same period period [101]. The set of these data suggests the bacterial resistance may explains the outcome of Gram-negative episodes treated with aminoglycosides.

The superiority observed with a glycopeptide plus ceftazidime must be carefully examined, because only 443 peritonitis episodes, in four case series [60–63] and only one RCT [59] were given this treatment. In addition, the comparisons among aminoglycosides, ceftazidime and fluoroquinolones used for the treatment of Gram-negative bacilli showed no differences in the resolution rates. Although the majority of these studies did not report the description of the bacterial resistance profile, differences in resistance may have influenced the outcome.

The present study confirms previous findings that showed no differences between vancomycin and first generation cephalosporins for the treatment of Gram-positive cocci. However, it should be considered that an increase in methicillin-resistant coagulase negative staphylococci as causal agents of PD-related peritonitis has been reported by several authors [75, 102], and that the results of this review may reflect conditions associated with the era or specific characteristics of each center.

This review has several limitations. The most important is the lower evidence level of case studies compared with the study designs of studies included in traditional systematic reviews. In addition, our analysis shows that there is significant heterogeneity in resolution rate. Finally, the studies differed considerably in their patient selection, baseline renal diseases, number of subjects, antibiotic administration routes, and other aspects. In conclusion, this review showed that the protocol of a glycopeptide plus ceftazidime could be a promising initial therapy in patients with PD-related peritonitis. This result should be carefully analyzed, and an emphasis should be placed on the necessity of monitoring the local microbiologic profile in each center regarding the initial therapeutic choice.

## Conclusion

The association of a glycopeptide plus ceftazidime was superior to other regimens for initial treatment of PD peritonitis. This result should be carefully analyzed and does not exclude the necessity of monitoring the local microbiologic profile in each dialysis center to choice the initial therapeutic protocol.

## Appendix

Summary of the bibliographic search strategies for type of clinical situation and intervention of interest.

[(Primary Peritonitis) OR (Secondary Peritonitis) OR (Peritoneal Dialyses) OR (Peritoneal Dialyses) OR CAPD OR (Continuous Ambulatory Peritoneal Dialysis) OR APD OR (Automated Peritoneal Dialysis)] AND [(Anti Bacterial Agents) OR (Antibacterial Agents) OR (Anti-Mycobacterial Agents) OR (Anti Mycobacterial Agents) OR (Antimycobacterial Agents) OR Antibiotic OR Antibiotics OR (Bactericidal Agents) OR Bactericides).

## Authors’ original submitted files for images

Below are the links to the authors’ original submitted files for images. Authors’ original file for figure 1 Authors’ original file for figure 2 Authors’ original file for figure 3 Authors’ original file for figure 4 Authors’ original file for figure 5 Authors’ original file for figure 6 Authors’ original file for figure 7 Authors’ original file for figure 8
