# Supplementary figures and images for: Efficacy of antibiotic therapy for peritoneal dialysis-associated peritonitis: a proportional meta-analysis
Source: BMC Infect Dis. 2014 Aug 18;14:445. doi: 10.1186/1471-2334-14-445 (PMC4262222; doi:10.1186/1471-2334-14-445)

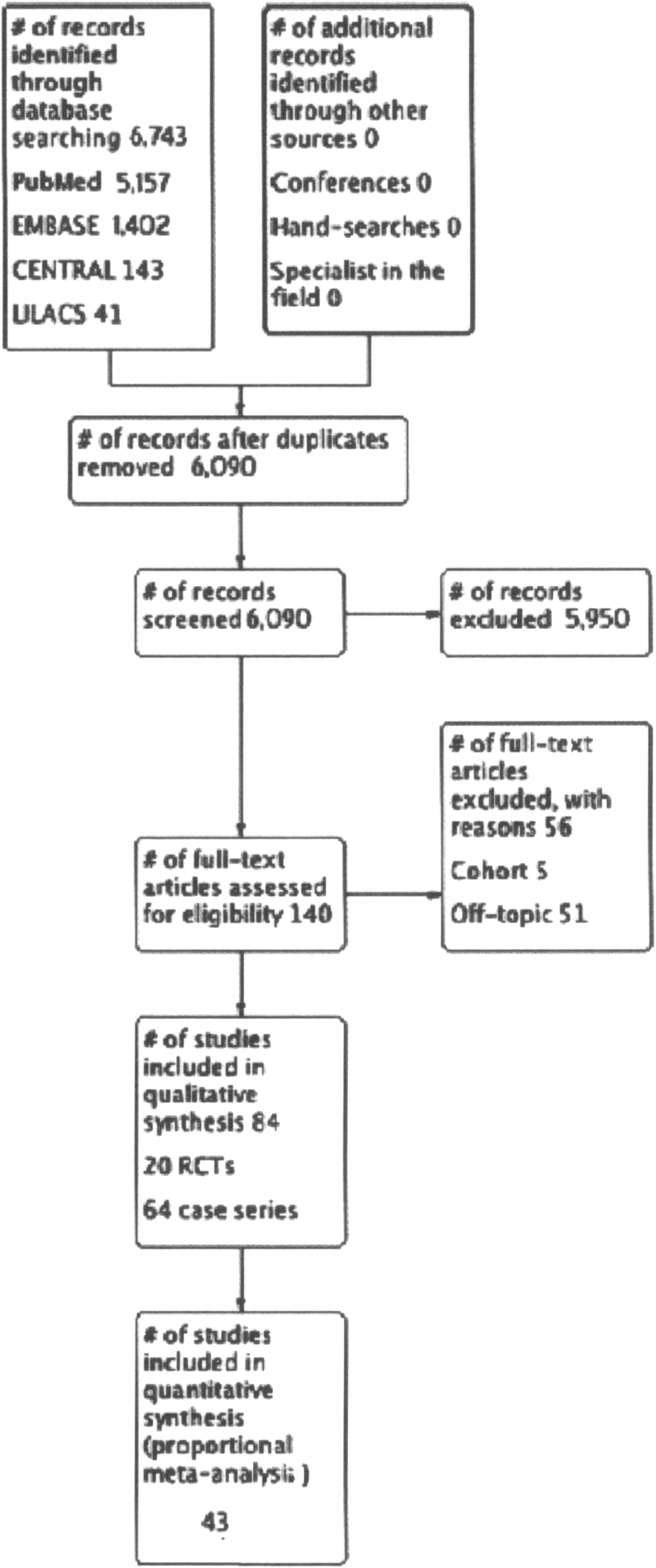

Supplement: Supplementary file 1 — Authors’ original file for figure 1 [file 12879_2013_4051_MOESM1_ESM.tiff]

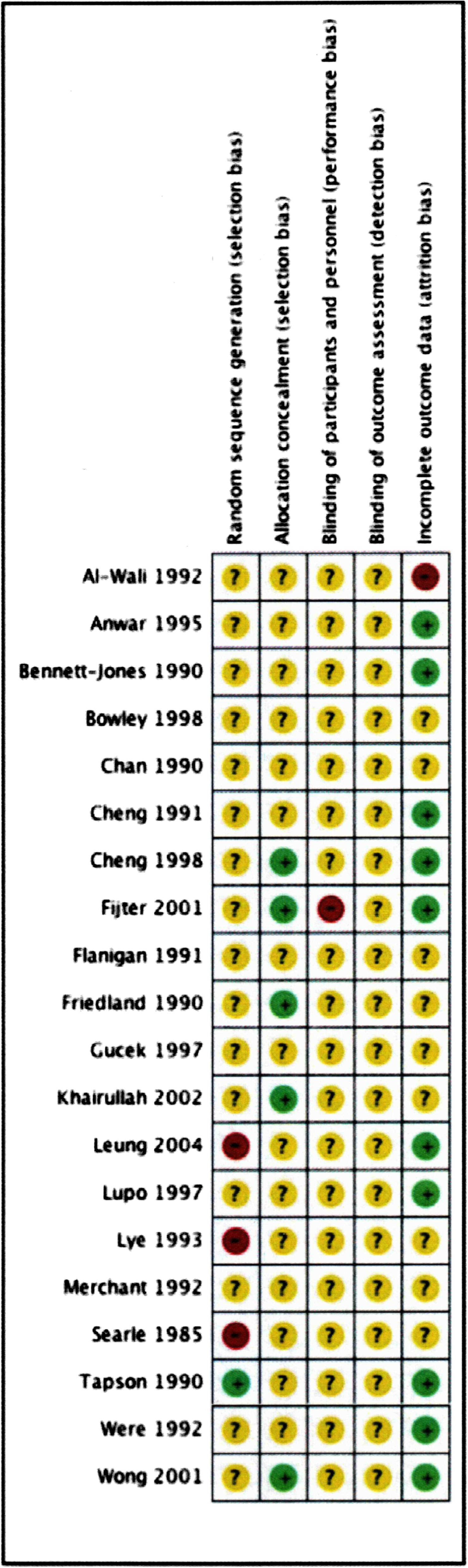

Supplement: Supplementary file 2 — Authors’ original file for figure 2 [file 12879_2013_4051_MOESM2_ESM.tiff]

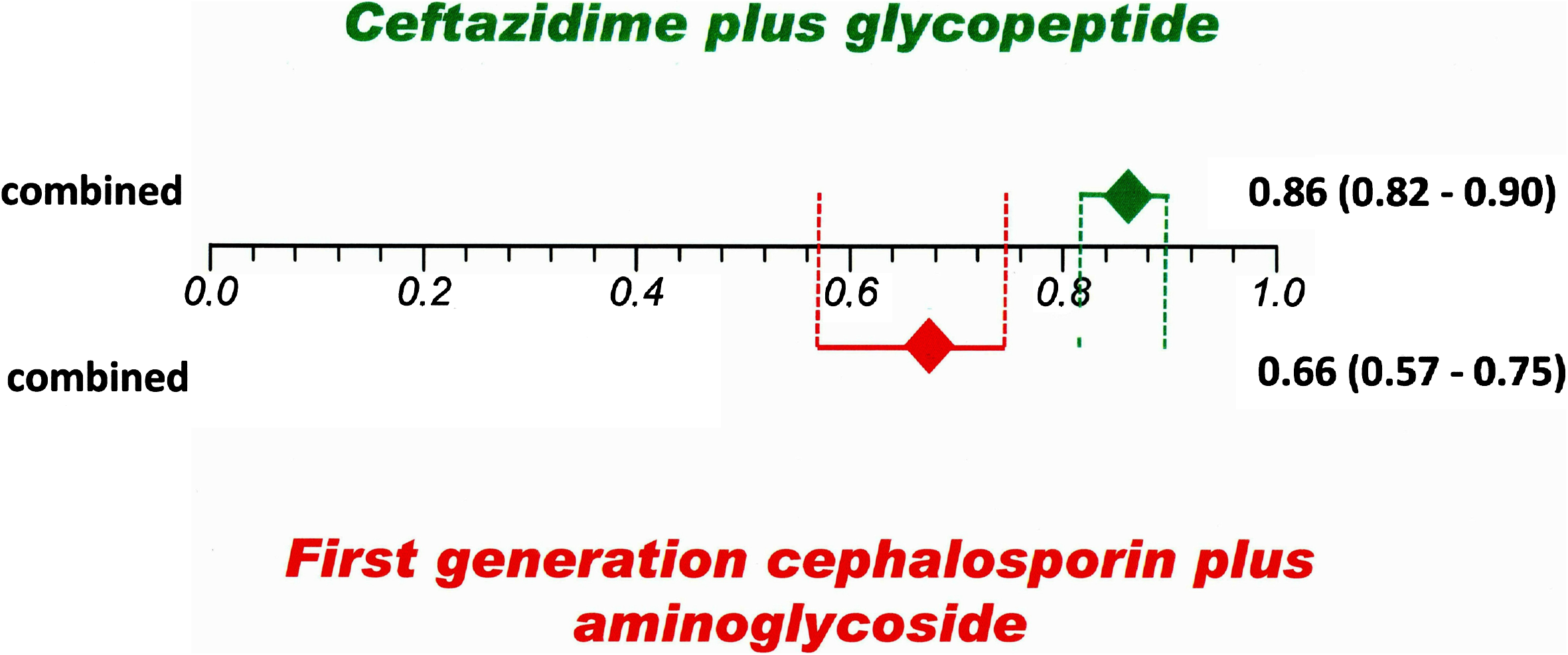

Supplement: Supplementary file 3 — Authors’ original file for figure 3 [file 12879_2013_4051_MOESM3_ESM.tiff]

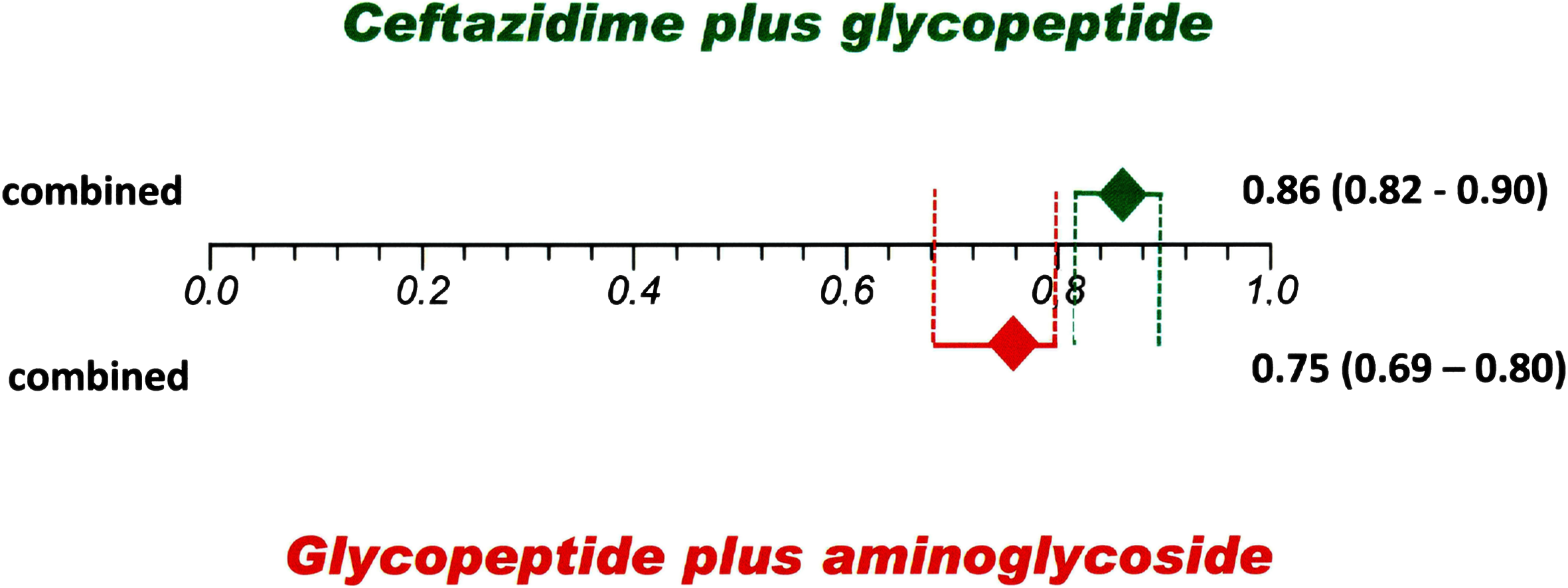

Supplement: Supplementary file 4 — Authors’ original file for figure 4 [file 12879_2013_4051_MOESM4_ESM.tiff]

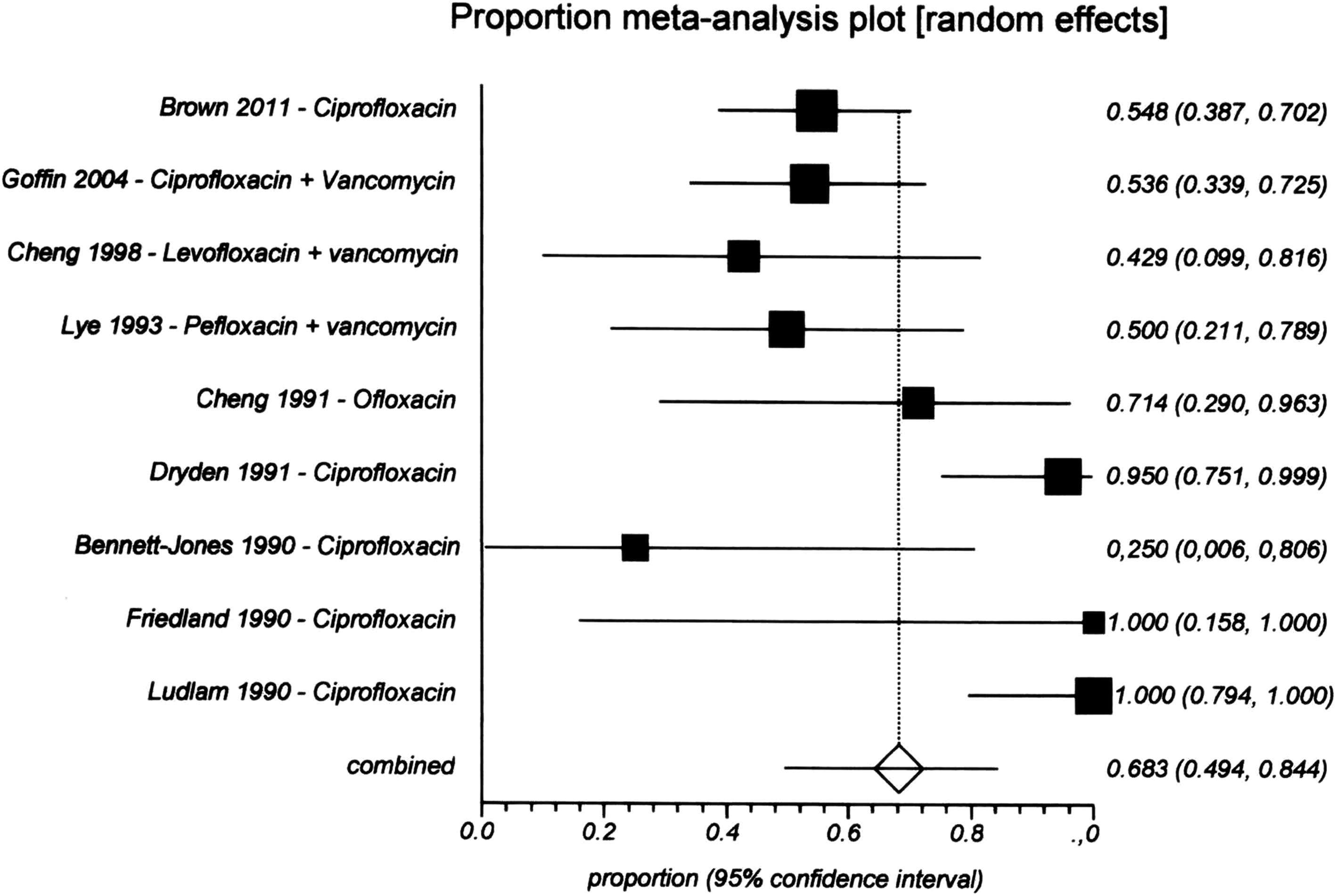

Supplement: Supplementary file 5 — Authors’ original file for figure 5 [file 12879_2013_4051_MOESM5_ESM.tiff]

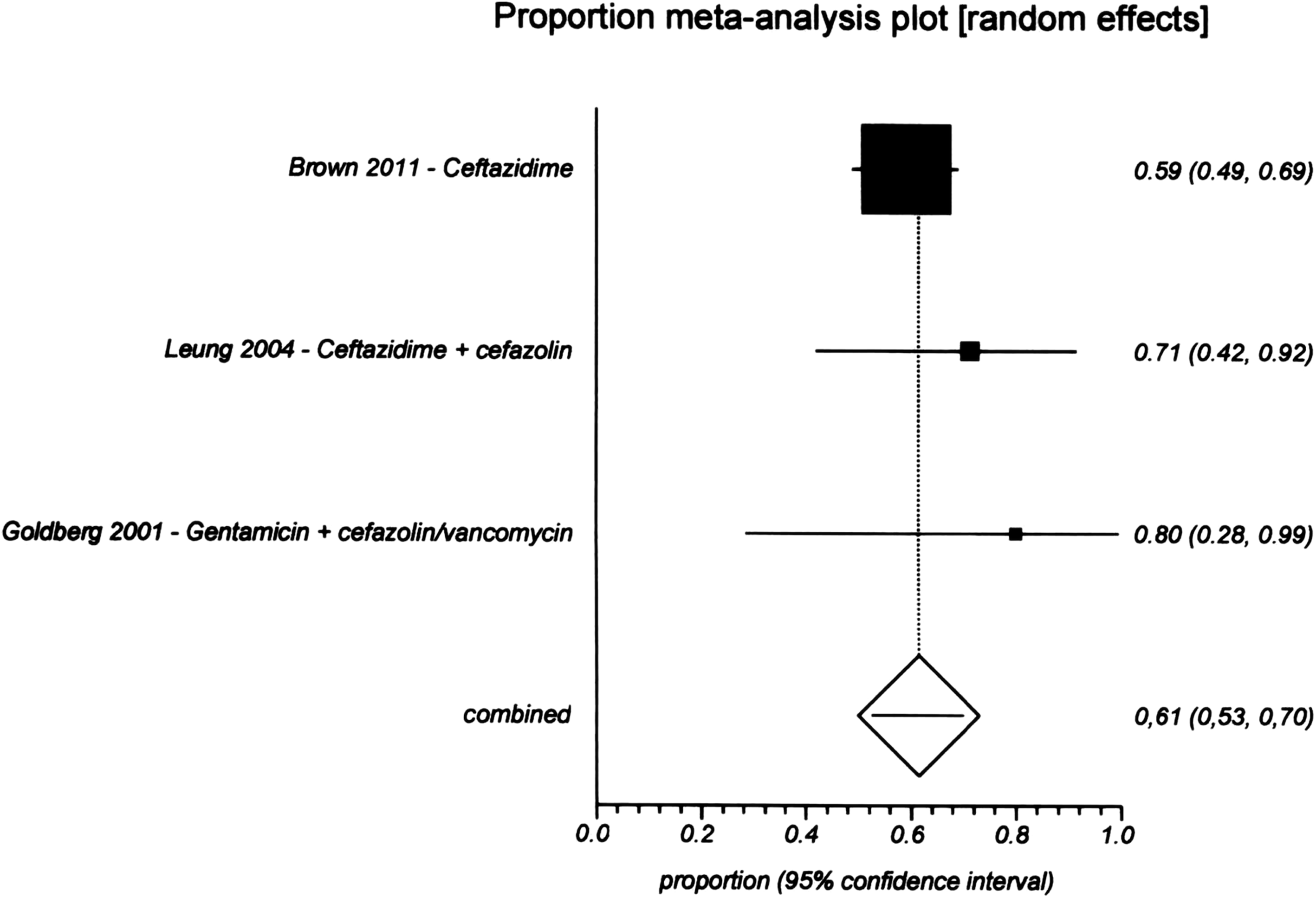

Supplement: Supplementary file 6 — Authors’ original file for figure 6 [file 12879_2013_4051_MOESM6_ESM.tiff]

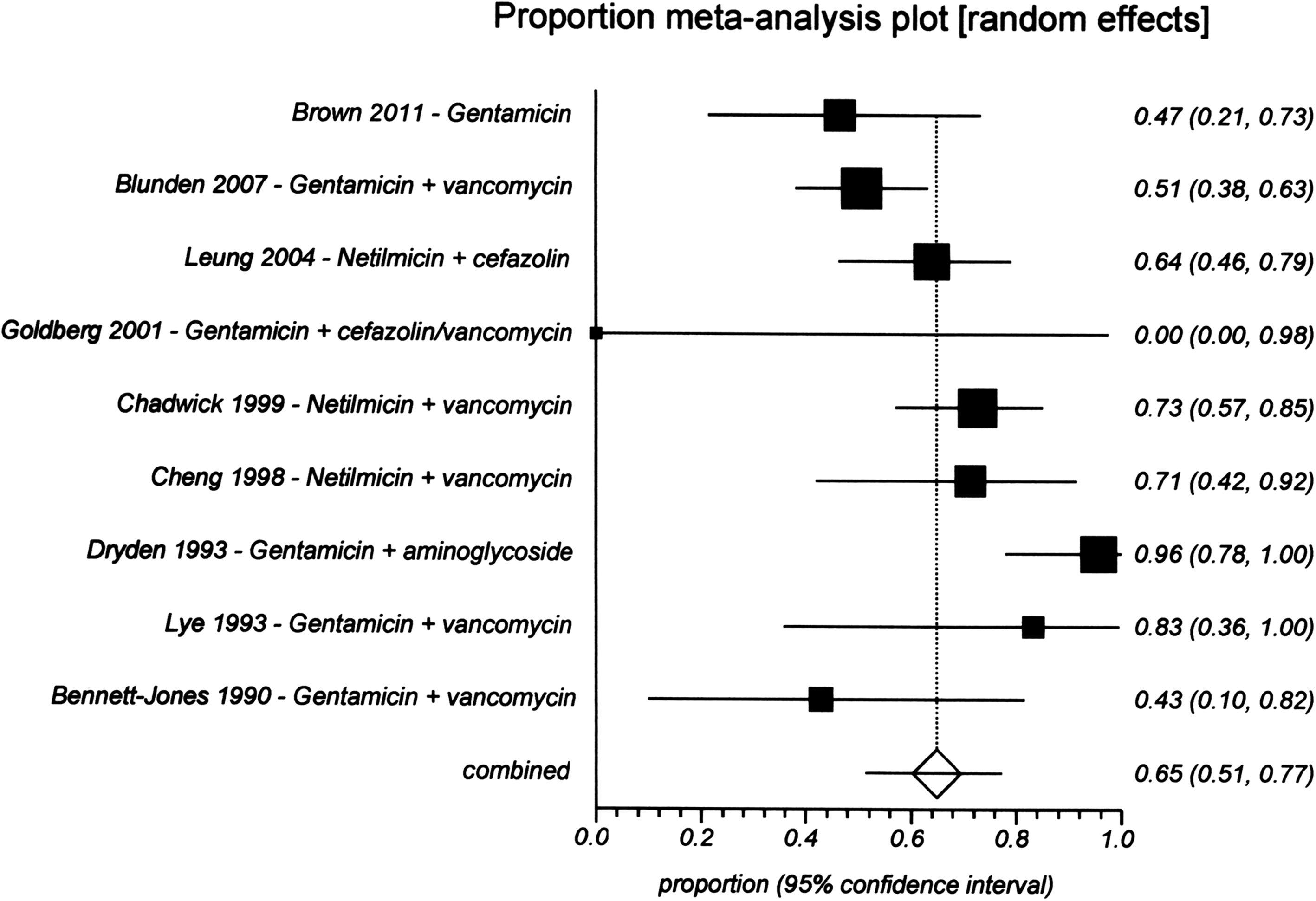

Supplement: Supplementary file 7 — Authors’ original file for figure 7 [file 12879_2013_4051_MOESM7_ESM.tiff]
